# A Preliminary Study for Distinguish Hormone-Secreting Functional Adrenocortical Adenoma Subtypes Using Multiparametric CT Radiomics-Based Machine Learning Model and Nomogram

**DOI:** 10.3389/fonc.2020.570502

**Published:** 2020-09-29

**Authors:** Yineng Zheng, Xin Liu, Yi Zhong, Fajin Lv, Haitao Yang

**Affiliations:** Department of Radiology, The First Affiliated Hospital of Chongqing Medical University, Chongqing, China

**Keywords:** radiomics, machine learning, multidetector computed tomography, computer-assisted diagnosis, adrenocortical adenoma

## Abstract

**Purpose:** To explore the application value of multiparametric computed tomography (CT) radiomics in non-invasive differentiation between aldosterone-producing and cortisol-producing functional adrenocortical adenomas.

**Methods:** This retrospective review analyzed 83 patients including 41 patients with aldosterone-producing adenoma and 42 patients with cortisol-producing adenoma. The quantitative radiomics features were extracted from the complete unenhanced, arterial, and venous phase CT images. A comparative study of several frequently used machine learning models (linear discriminant analysis, logistic regression, random forest, and support vector machine) combined with different feature selection methods was implemented in order to determine which was most advantageous for differential diagnosis using radiomics features. Then, the integrated model using the combination of radiomic signature and clinic–radiological features was built, and the associated calibration curve was also presented. The diagnostic performance of these models was estimated and compared using the area under the receiver operating characteristic (ROC) curve (AUC).

**Result:** In the radiomics-based machine learning model, logistic regression model with LASSO (least absolute shrinkage and selection operator) outperformed the other models, which yielded a sensitivity of 0.935, a specificity of 0.823, and an accuracy of 0.887 [AUC = 0.882, 95% confidence interval (CI) = 0.819–0.945]. Moreover, the nomogram representing the integrated model achieved good discrimination performances, which yielded a sensitivity of 0.915, a specificity of 0.928, and an accuracy of 0.922 (AUC = 0.902, 95% CI = 0.822–0.982), and it was better than that of the radiomics model alone.

**Conclusion:** This study found that the combination of multiparametric radiomics signature and clinic–radiological features can non-invasively differentiate the subtypes of hormone-secreting functional adrenocortical adenomas, which may have good potential for facilitating the diagnosis and treatment in clinical practice.

## Introduction

Adrenocortical adenomas (ACAs) are the most common benign adrenal cortical tumors representing 50–80% of all adrenal tumors (1) that may be functional (hormone-secreting) or non-functional depend on whether producing hormones. Among functioning adenomas, two major subtypes are aldosterone-producing adenoma (APA) and cortisol-producing adenoma (CPA), leading to respective complications including primary aldosteronism (Conn syndrome) and hypercortisolism (Cushing syndrome), and each requires different treatment strategies including surgery or medications. The diagnosis of the functional ACAs is dependent on the clinical manifestations, laboratory tests, imaging, and pathologic examinations forming the basis to conclude. However, the differential diagnosis between APA and CPA still remains challenging because many patients are asymptomatic or there were only non-specific symptoms with no clinical evidence of steroid overproduction (2, 3). The gold standard for the diagnosis of APA is through a technically difficult and invasive procedure that samples from a vein located near the adrenal glands, called adrenal vein sampling (AVS), to determine aldosterone and cortisol levels (4, 5) The success rate of right AVS is as low as 10% because of the particular and complex anatomical structure (6). Moreover, about 10–20% of ACAs are bilateral or multiple (7, 8). In such condition, it is very important, but also quite difficult, to distinguish the responsible foci to avoid unnecessary excision or overresection for performing precision treatment.

In general, current conventional imaging methods are insufficient to distinguish between functioning and non-functioning adenomas or subtypes of functioning ACAs. As an emerging medical image processing technology, radiomics provides the potential for more refined representation of tumor characteristics with isotropic homogeneity and leads to the advantage over human observers, which have demonstrated promising performance in terms of differential diagnosis. It has been proven that radiomics procedure can process a large number of image characteristics and implement automatic diagnostic process (9, 10), combining with machine learning algorithms and nomogram method (11). To our knowledge, little work has been done on such a computed tomography (CT)–based radiomics to distinguish CPA from APA, and whether radiomics features of CT images can serve as the informative biomarkers for the differential diagnosis between those is unknown.

With this in mind, we conducted two hypotheses. One was that the radiomics-based machine learning model could provide a computer-aided differential diagnosis for hormone-secreting subtypes of functional ACAs; the other was that the nomogram that integrated radiomics signature and clinic–radiological indicators would improve the differential diagnostic performance. Therefore, the primary purpose of our study was to determine whether multiparametric CT radiomics by using machine learning algorithms and visual nomogram effectively perform differential diagnosis between CPA and APA.

## Materials and Methods

**Figure 1:**
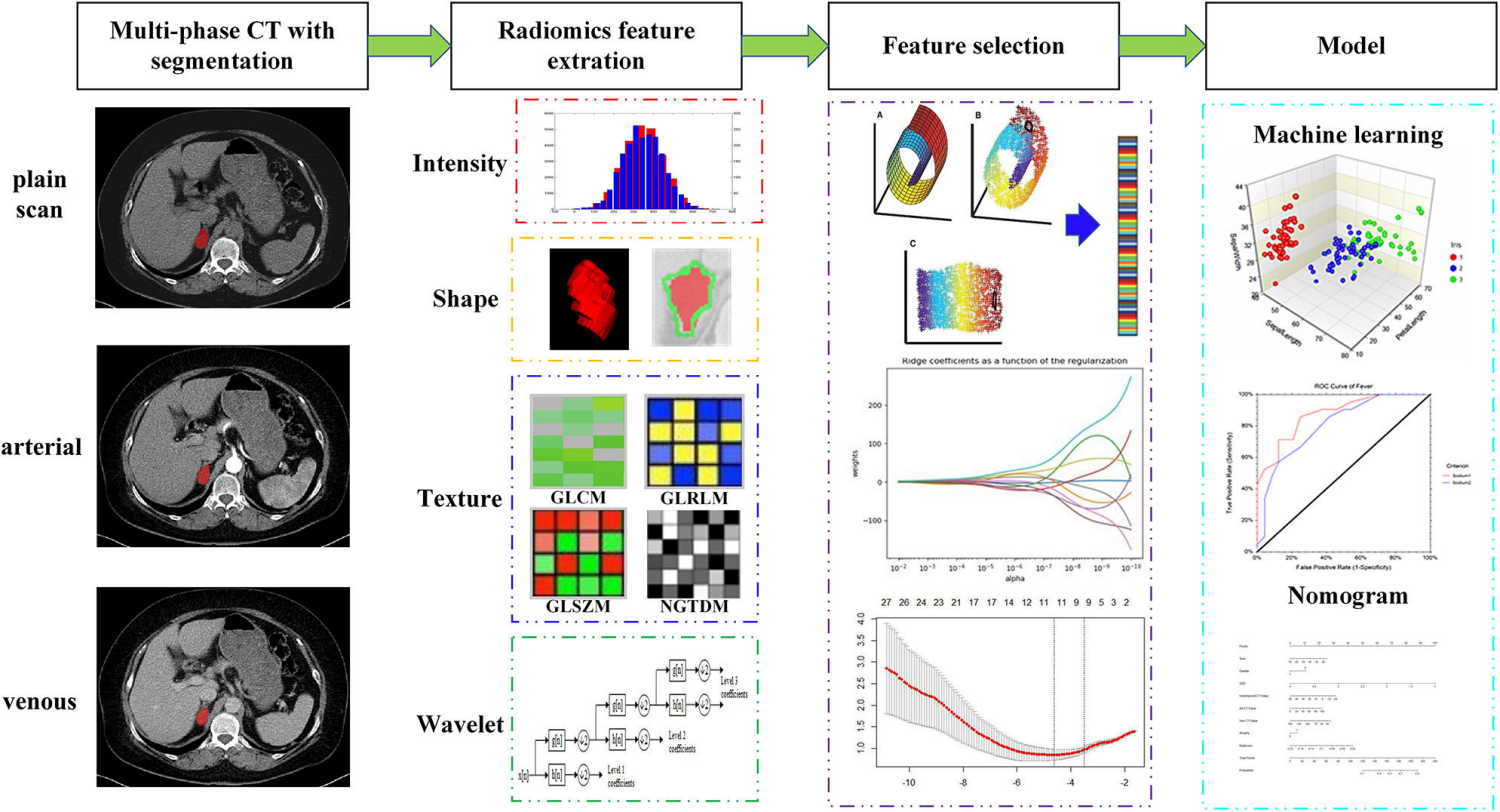
Workflow of multiparametric CT radiomics-based machine learning model and nomogram.

The research sequence of this study was presented in Figure 1. The details could be checked in the following sections.

### Profile of Subjects

**Figure 2:**
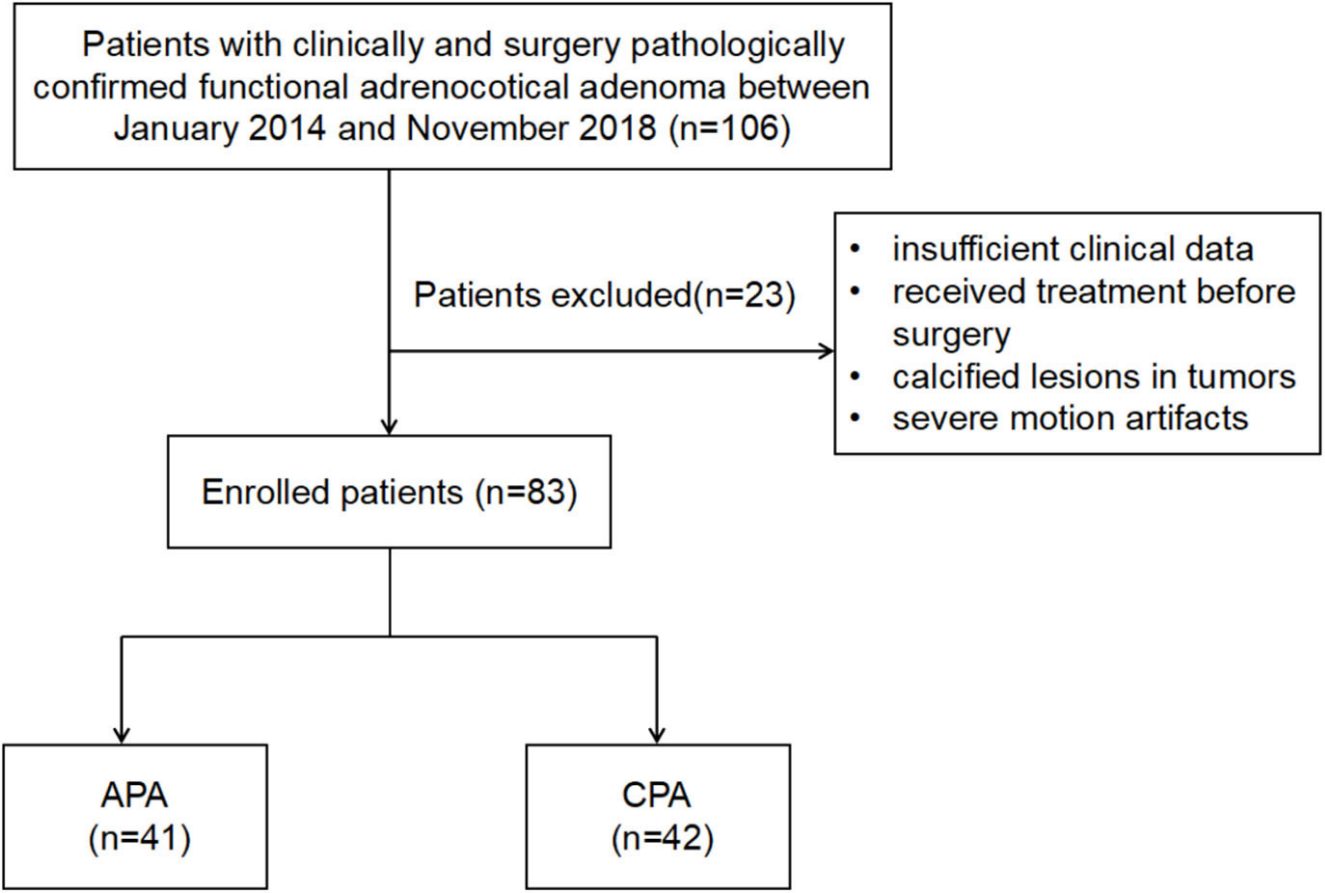
The illustration of inclusion and exclusion criteria.

This retrospective study was approved by the institutional review board of our institution, and the written informed consent was waived. The enrolment process of patients for this study is shown in Figure 2. In total, 106 patients who underwent contrast-enhanced CT scannings for clinically and pathologically diagnosed CPA (*n* = 50) or APA (*n* = 56) from January 2014 to November 2018 were retrospectively reviewed. The diagnosis of APA and CPA was established by these criteria: (i) common clinical characteristics and laboratory findings including an elevated aldosterone/renin ratio together with positive confirmatory tests in APA and an elevated serum cortisol, failure to suppress cortisol with dexamethasone, and normal aldosterone levels in CPA, respectively; (ii) presence of an adrenal mass confirmed via CT before surgery; (iii) a confirmed pathological diagnosis of the adrenal mass as an adrenal adenoma after surgery; and (iv) a postoperative cure or considerable improvement. The exclusion criteria were as follows: (i) insufficient clinical data; (ii) receiving treatment before surgery; (iii) calcified lesions in tumors; and (iv) motion artifacts disturbed the lesion characterization severely. Finally, 9 patients with CPA and 14 patients with APA were excluded. The dataset was divided into two portions called training set and testing set, 70% of which were used as training set, and the remaining 30% were used as test set.

### Imaging Protocol

All CT imaging was acquired using the same multidetector CT system (Somatom Sensation 64; Siemens Healthcare, Erlangen, Germany) following a standardized protocol. A three-phase scanning was performed on each patient (plain scan, arterial phase, and venous phase). The CT scanning parameters were as follows: tube voltage of 100 kV; tube current of 75 mAs, and slice thickness of 5 mm. Images were reconstructed using a B60f filter with a slice thickness of 1 mm and a slice increment of 1 mm as axial images. Contrast-enhanced CT images were obtained after intravenous administration of iohexol (300 mg/mL at a rate of 3.0 mL/s, followed by a 30-mL saline flush). Arterial phase imaging and portal phase imaging were initiated at 30 and 70 s after the injection of iohexol. The total contrast volume was 1.5 mL/kg.

### Imaging Segmentation and Volume of Interest Labeling

**Figure 3:**
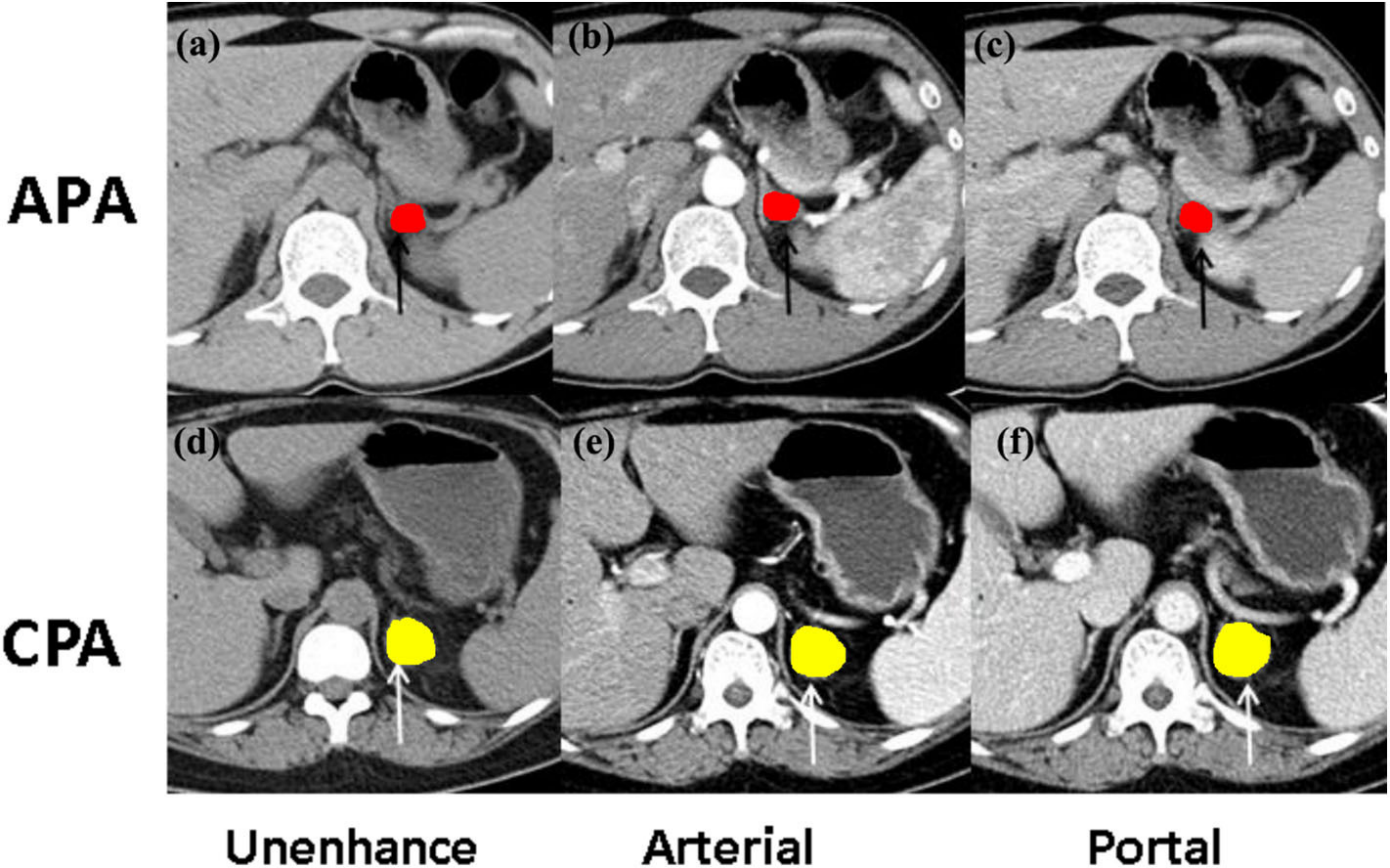
The illustration of ROI selection. **(a)** The Unenhanced and contrast-enhanced CT images at **(b)** the arterial phase and **(c)** portal phase of CT imaging findings in a 46-year-old woman with aldosterone-producing adenoma (APA, black arrow); **(d)** the unenhanced and contrast-enhanced CT images at **(e)** the arterial phase and **(f)** portal phase of CT imaging findings in a 39-year-old man with cortisol-producing adenoma (CPA, white arrow).

All CT images (DICOM format) were loaded into a computer workstation for region of interest (ROI) segmentation, which were displayed with the appropriate window level and window width. Two radiologists with 5-year experience in interpreting CT imaging (Dr. Liu and Zhong) were recruited to manually delineate the two-dimensional (2D) ROI around the tumor outline slice by slice to form 3D volume of interest (VOI) on the CT plain scan, arterial phase, and venous phase images using an open-source image processing platform ITK-SNAP (version 3.7) (12), where horizontal, coronal, and sagittal views were represented simultaneously for visualization. Image magnification and 3D view techniques have been used to facilitate precise segmentation (Figure 3).

### Imaging Analysis

Conventional imaging analysis was included the following information: (a) tumor size; (b) mean CT attenuation of tumor in precontrast, arterial, and portal venous phase; and (c) the presence of ipsilateral or contralateral adrenocortical atrophy. The mean CT attenuation was used to describe average value of tumor density over the CT pixels and automatically obtained by drawing ROI around tumor contour on workstation. The presence of adrenocortical atrophy was defined as the maximum thickness of a unilateral adrenal gland more than a 50% reduction compared to the other side.

### Radiomics Feature Extraction

The radiomics features were extracted from each VOI segmentation derived from multiparameter CT images, which are divided into four feature groups: (I) intensity, (II) shape, (III) texture, and (IV) wavelet features (11, 13). Supplementary Tables 1–3 summarize these features in this study. Mathematical definitions of all radiomics features, as well as the extraction methods, have been described (14). The texture features were computed by averaging their values over all 13 directions. Wavelet features are the transformed domain representations of the intensity and textural features, which were computed on different wavelet decompositions of the original image using a Daubechies wavelet transformation. Finally, the combination of four categories of features derived from multiphase CT images was incorporated into the radiomics feature set.

### Reproducibility Evaluation and Feature Selection

Radiomics feature reproducibility was evaluated prior to feature selection by computing the intraclass correlation coefficient (ICC). Each radiomics feature with ICC more than 0.8 was set to consider robust to acquisition variation (15) (see Supplementary), which were retained based on the hypothesis that non-robust features would be too sensitive to noise to be predictive of clinical outcomes. Feature selection as an important problem for pattern classification has become an apparent need in the radiomics application. To find optimal characterization condition and achieve minimal classification error in machine learning, feature relevancy needs to be eliminated. The extracted radiomics features were selected using principal component analysis (PCA), ReliefF algorithm, least absolute shrinkage and selection operator (LASSO), recursive feature elimination, and mutual information. We chose these methods mainly because of their popularity, simplicity, and computational efficiency. All features have been normalized to zero mean and unit variance so as to avoid being affected by the differences in respective feature scales for classification model building. Furthermore, publicly available implementations were readily available for these methods, which increases their reusability.

### Construction of the Radiomics-Based Machine Learning Model

For the model development, four different algorithms such as linear discriminant analysis, logistic regression (LR), random forest, and support vector machine (SVM) have been adopted. Each classifier has been tested and verified using the feature sets obtained by the different feature selection methods to construct the stable and optimal machine learning model. In the training set, efficient data partitioning such as 5-fold cross-validation was employed to tune and optimize the model parameter to achieve good assessment of the model performance (16). The area under the receiver operating characteristic (ROC) curve (AUC), sensitivity, specificity, and accuracy were used as metrics to assess the performance of the machine learning models. All classifier algorithms were implemented by our in-house scripts in MATLAB (version 2017b, MathWorks, Natick, MA, USA).

### Establishment and Validation of the Nomogram

The nomogram was used to represent the integrated model for distinguishing APA from CPA. The radiomics signature was constructed by the selected features sorted by their coefficient values in LASSO. Then, the nomogram based on the multivariate logistic analysis was developed by using the combination of radiomics signature and clinic–radiological features as a quantitative diagnostic tool to provide physicians with an individual prediction probability of APA. Calibration curves accompanied by the Hosmer–Lemeshow test were used to assess the model performance. AUC, accuracy, PPV, and NPV were calculated to quantify the diagnostic performance of nomogram. The 1,000-bootstrap repetitions were carried out for internal validation to achieve a relatively corrected performance where the training cohort was randomly chosen with a replacement from the original dataset.

### Statistics

Continuous variables, expressed as mean value ± standard deviation or median with interquartile range as appropriate, were analyzed using Student *t*-test or Mann-Whitney *U*-test, respectively. Categorical/dichotomous variables, expressed as counts (percentage), were analyzed using a χ^2^-test or Fisher exact test as appropriate. Multiple and pairwise comparisons of AUCs were accomplished using the DeLong non-parametric approach. Univariate and multivariate logistic regression models were employed to select the independent clinical features and construct clinic–radiological model. Statistical analysis was performed with R version 3.6.1 (http://www.r-project.org). A two-sided *p* < 0.05 was considered to represent statistically significant.

## Results

### CT Findings and Clinic–Radiological Model

The demographic data and radiological characteristics between APA and CPA are presented in Table 1. Sex ratio and age distribution did not differ significantly between these two groups (*p* > 0.05). In conventional CT findings analysis, there were significant differences between CPA and APA groups in tumor size, mean CT attenuation value of precontrast phase and portal venous phase, and the presence of adrenocortical atrophy (*p* < 0.05). In the APA group, the tumor showed smaller size and lower mean CT attenuation compared to CPA group, while the ipsilateral or contralateral adrenocortical atrophy was more commonly seen in CPA group (Table 2).

**Table 1:** Demographic data of this study.

**Type**	**APA**	**CPA**	***P***
Number of patients	41	42	
Age (years)	46.8 ± 7.95	47.9 ± 8.17	0.469
Sex	Female (24)	Female (30)	0.316
	Male (17)	Male (12)	

**Table 2:** Clinical characteristics and CT findings of patients with CPA and APA.

**Characteristics**	**Training cohort (*****n*** **=** **58)**		***p***	**Test cohort (*****n*** **=** **25)**		***p***
	**APA (*n* = 29)**	**CPA (*n* = 29)**		**APA (*n* = 12)**	**CPA (*n* = 13)**	
Age (years)^#^	45.9 (8.9)	51 (11.6)	0.008	49.0 (9.3)	41.1 (10.9)	0.018
Sex^*^						
Female	17 (58.6)	21 (72.4)	0.407	7 (58.3)	9 (69.2)	0.057
Male	12 (41.4)	8 (27.6)		5 (41.7)	4 (30.8)	
Size^#^	1.6 (0.54)	2.64 (1.57)	0.001	1.5 (0.61)	2.62 (0.40)	0.025
Unenhanced^#^	5.9 (10.3)	9.31 (16.3)	<0.01	6.3 (9.2)	13.9 (16.4)	<0.001
Art^#^	47.4 (17.6)	50.6 (24.9)	0.002	48.9 (19.7)	45.8 (24.9)	0.061
Ven^#^	56.7 (17.8)	64.5 (23.5)	<0.01	57.5 (17.2)	79.5 (35.9)	<0.001
Atrophy^*^						
Yes	4 (13.8)	11 (37.9)	0.071	1 (8.3)	7 (53.8)	<0.001
No	25 (86.2)	18 (62.1)		11 (91.7)	6 (46.2)	

# *Data are mean (standard deviation) or median (quartile). p-value was calculated with Student t-test or non-parametric test*.

* *Data are number of patients, with the percentage in parentheses. p-value was calculated with the χ^2^ or Fisher exact test*.

In total, 58 patients including 29 patients with CPA and 29 patients with APA comprised the training cohort, and 25 patients including 12 patients with CPA and 13 patients with APA comprised the test cohort. The proportions of training cohort and the test cohort were 70 and 30%, respectively, and no significant differences of clinical characteristics or CT findings were found between the training and test cohorts (*p* > 0.05). Table 2 showed the significant differences between CAP and APA in the training and test cohorts.

### Radiomics Features Calculation and Robustness Assessment

The longest diameters and the ratio of longest diameter to shortest diameter of raw ROIs between two groups were distributed without statistical significance (*p* > 0.05), each of which was selected from the slice demonstrating the largest cross-sectional area on CT images. ICCs on the basis of radiologist I's first-extracted features and those of radiologist II were employed to evaluate the consistency between different physicians. The ICCs on the basis of radiologist I's first and second feature extraction were calculated to evaluate the stability and reproducibility of each feature. According to the criterion of excluding the radiomics features with ICC below 0.8, a total of 39 radiomics features were considered as robust shown in Supplementary Table 4.

### Radiomics Feature Selection and Machine Learning Model Performance Comparison

**Figure 4:**
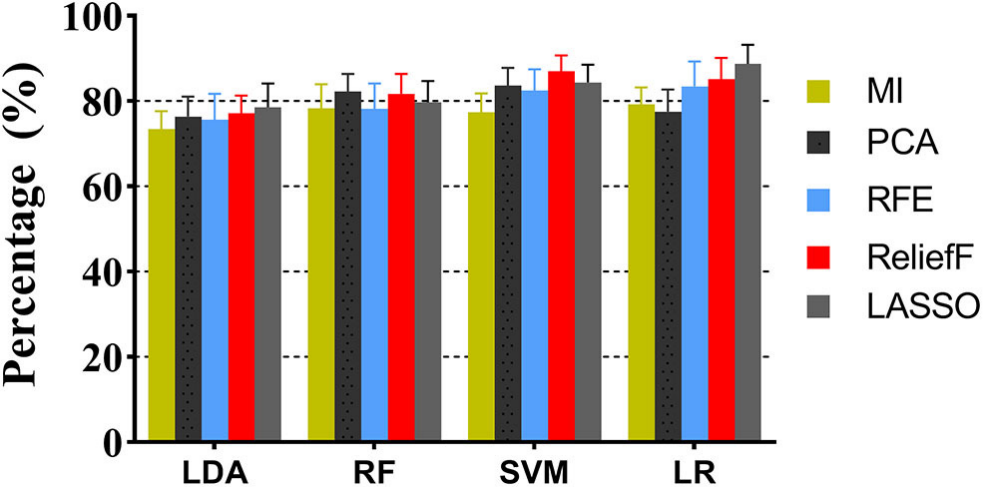
The performance comparison of machine learning models with different feature selection methods.

Feature selection determines the minimum set of relevant indicators needed by a machine learning model. The above robust radiomics features are further screened by retaining those that differed significantly between the two groups. Twenty classification strategies using combinations of four machine learning and five feature selection methods, respectively, have been tested, and the AUCs for differential diagnosis between CPA and APA in the test dataset are shown in Figure 4. For the combination of multiple sequences, it is shown that the LR combined with LASSO performs better and more stable than the other models, which yielded a sensitivity of 0.935, a specificity of 0.823, and an accuracy of 0.887 [AUC = 0.882, 95% confidence interval (CI) = 0.819, 0.945], followed by SVM classifier with ReliefF, yielding an accuracy of 0.842 (AUC = 0.854, 95% CI = 0.811–0.897) in the test cohort.

### Performance of Sequences

**Figure 5:**
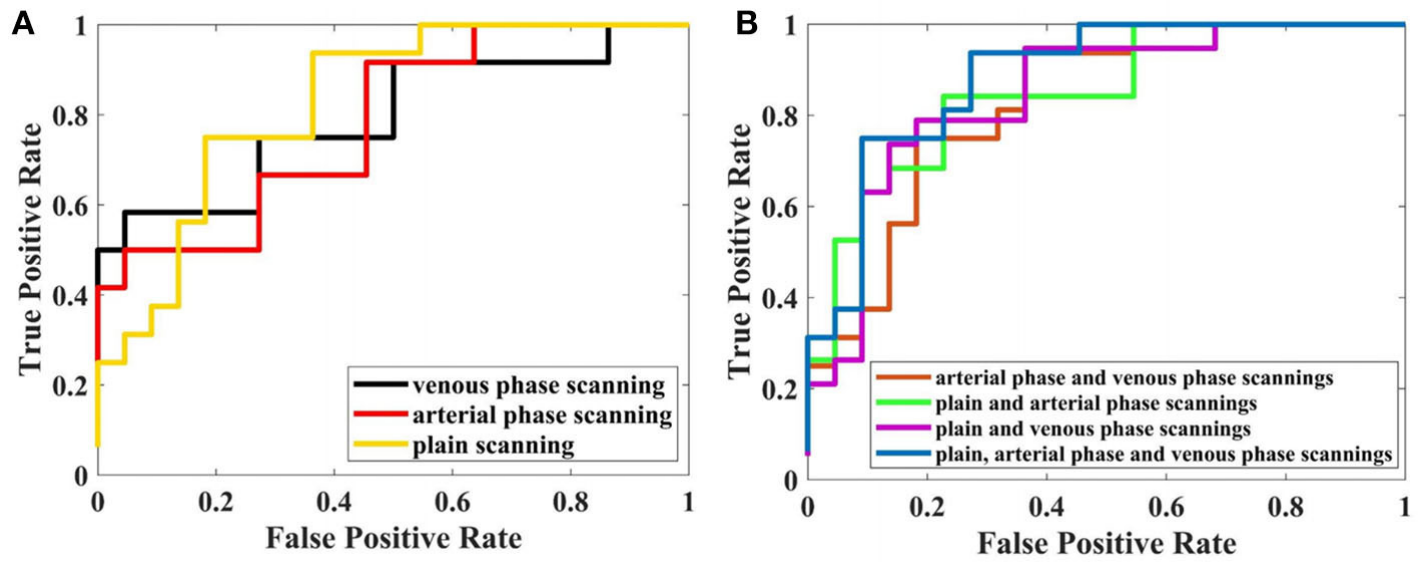
Performance of LR-LASSO model based on different sequences with 5-fold cross-validation. **(A)** Based on single sequence. **(B)** Based on multiparametric CT.

The discriminative performance of the LR-LASSO models using the radiomics features from multiple sequences and single sequence was investigated. The ROC curves for all single sequences such as CT plain, arterial phase, and venous phase scanning are shown in Figure 5A, and the ROC curves for the combination of multiple sequences are shown in Figure 5B. For single sequence, the performance of plain and venous phase scanning is similar, and the highest AUC was 0.834 (95% CI = 0.779–0.889). For two sequences, the performance of the combination of plain and venous phase scanning was highest with an AUC of 0.876 (95% CI = 0.808–0.944). For three sequences, the model performed best and yielded the highest AUC of 0.882 (95% CI = 0.819–0.945). The AUCs among the three single sequences were not statistically significant, while the DeLong test showed that the AUCs for the different combinations of the multiple sequences were significantly better than those of single sequences, and the AUC for the combinations of the three sequences was the highest.

### The Combined Model Incorporating Radiomics Signature and Clinic–Radiological Characteristics

**Figure 6:**
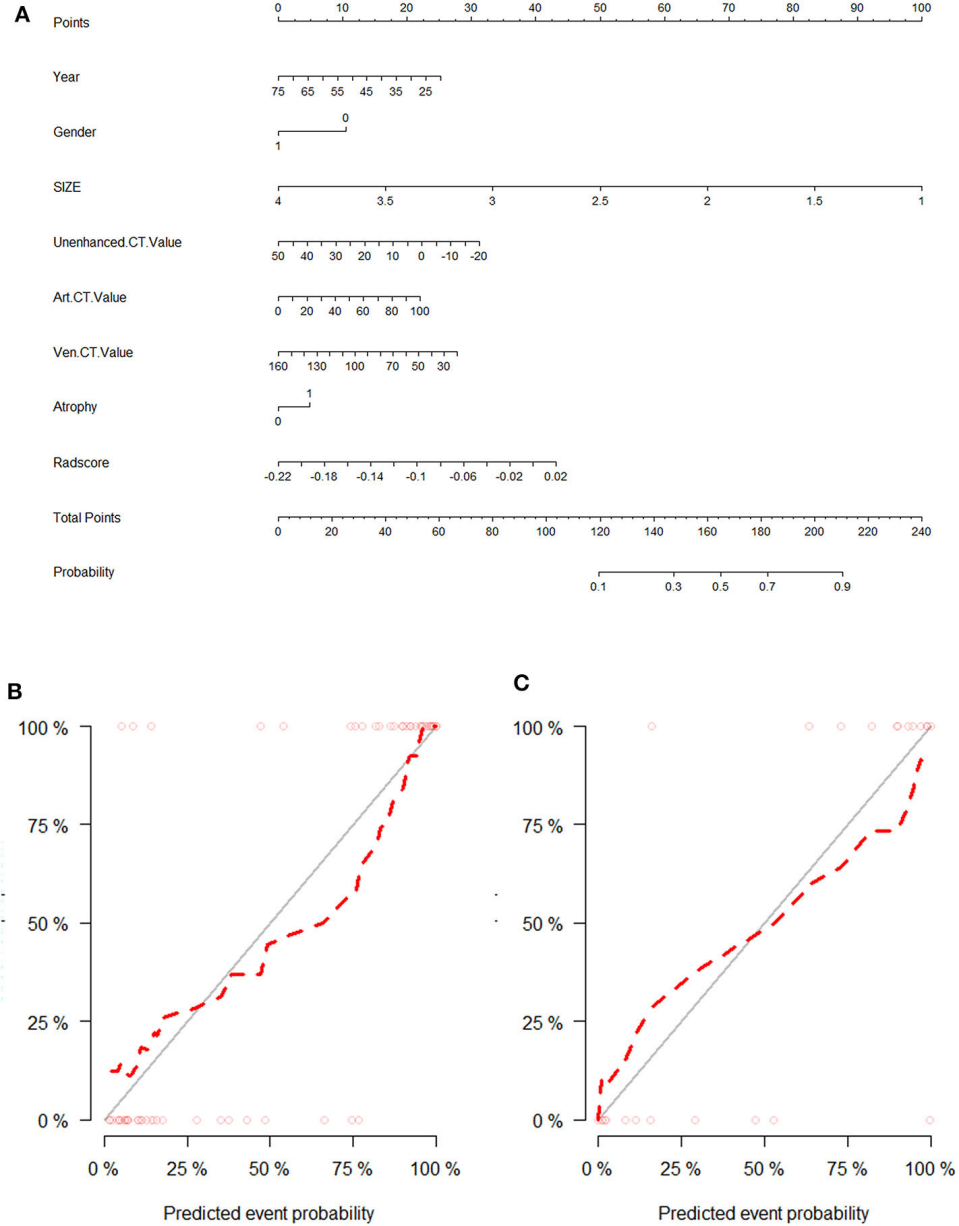
The visual presentation of nomogram combining the radiomics signature and clinic–radiological indicators **(A)** and its calibration curves in training cohort **(B)** and test cohort **(C)**.

**Figure 7:**
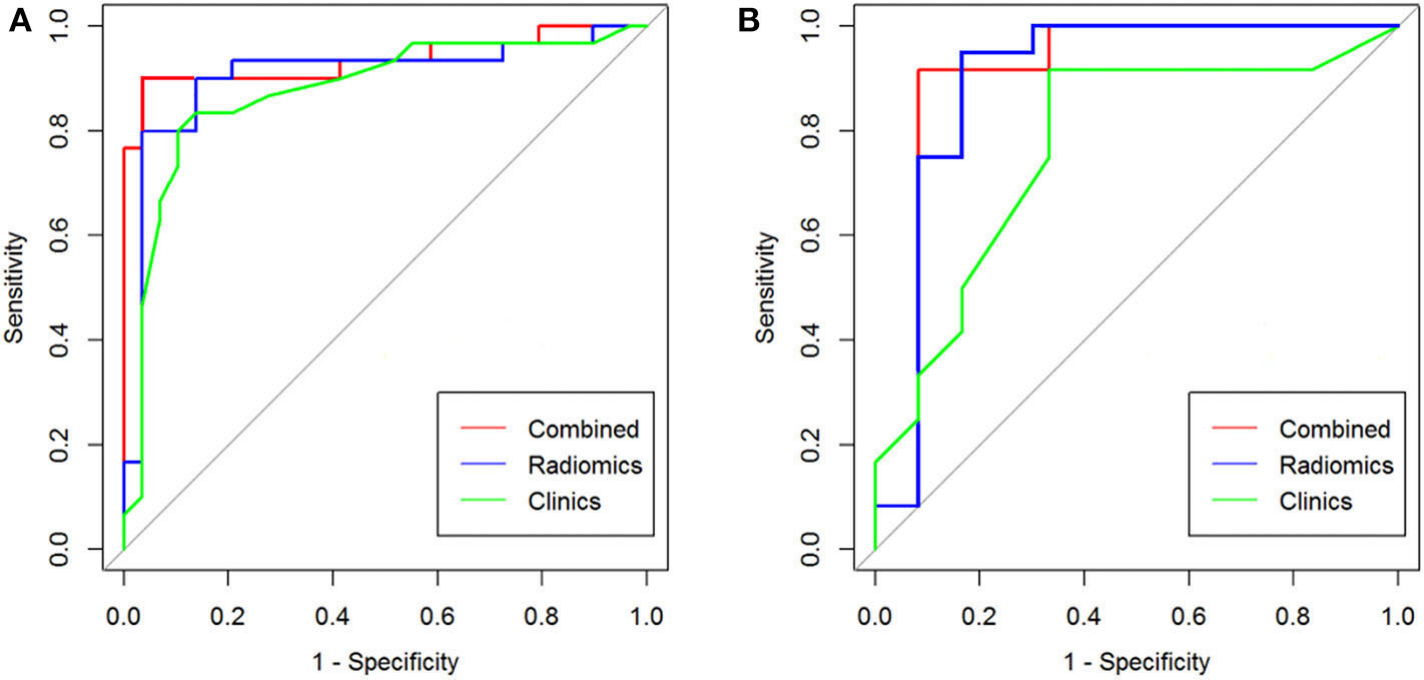
The ROC curve analysis for differential diagnostic efficiency of three models in training cohort **(A)** and test cohort **(B)**.

The above results revealed that the multiparametric CT radiomics-based LR-LASSO model would be most suitable to effectively differentiate CPA from APA. The clinic–radiological characteristics such as age, gender, tumor size, and CT value were determined to establish the clinical model. The combined model that incorporated radiomics signature and clinic–radiological characteristics was developed and presented as a radiomics nomogram (Figure 6A). The clinical model yielded an AUC of 0.829 (95% CI, 0.796–0.863) in the training cohort and 0.732 (95% CI, 0.671–0.793) in the test cohort. When clinic–radiological characteristics were combined, the radiomics nomogram yielded an AUC of 0.931 (95% CI = 0.869–0.993) in the training cohort and 0.902 (95% CI, 0.822–0.982) in the test cohort. Table 3, Figure 7 presented the detailed discrimination indicators of the three models. The calibration curves of the radiomics nomogram for differential diagnosis between CPA and APA showed good agreement between the model outcome and gold standard test in the training (Figure 6B) and test (Figure 6C) cohorts (*p* = 0.849 and 0.814, respectively; Hosmer-Lemeshow test). The net reclassification improvement (NRI) test showed the integrated model achieved considerably better discrimination ability than the clinic–radiological model (*p* = 0.012) and radiomics model (*p* = 0.012) in the training cohort. The performance of the integrated model was comparable to that of the radiomics model (*p* = 0.989; NRI test), but was superior to that of clinic–radiological model (*p* = 0.001; NRI test). The illustration in the supplementary material presented two cases pathologically diagnosed as CPA and APA, respectively, and the probability values predicted by the nomogram.

**Table 3:** Diagnosis performance of the three models.

**Model**	**Clinics**		**Radiomics**		**Nomogram**	
	**Training**	**Test**	**Training**	**Test**	**Training**	**Test**
AUC	0.829 (0.796, 0.863)	0.732 (0.671, 0.793)	0.897 (0.841, 0.953)	0.882 (0.819, 0.945)	0.931 (0.69, 0.993)	0.902 (0.822, 0.982)
Accuracy	0.745	0.714	0.881	0.887	0.933	0.922
Sensitivity	0.719	0.691	0.909	0.935	0.909	0.915
Specificity	0.798	0.735	0.876	0.823	0.968	0.928
PPV	0.801	0.746	0.869	0.836	0.951	0.931
NPV	0.723	0.687	0.914	0.933	0.914	0.907

*Data are percentages with 95% CIs in square parentheses. Nomogram indicates the integrated model combining of clinics and radiomics features; PPV, positive predictive value; NPV, negative predictive value*.

## Discussion

The prevalence of adrenal adenoma is reported to be related to age, and the frequency of unsuspected adenoma is 0.14% in patients aged 20–29 years and 7% in those older than 70 years (17). Most previous studies were concentrated on the imaging features of adenoma differentiated from other non-adenomas in patients such as hyperplasia, cyst, myelolipoma, pheochromocytoma, cortical carcinoma, and metastases (18). Recent investigations have revealed that multiple imaging modalities such as dual-energy CT, magnetic resonance (MR) chemical-shift imaging, diffusion-weighted imaging, MR spectroscopy, and dynamic contrast-enhanced imaging showed various sensitivity and specificity for differential diagnosis of adenoma (19–21). However, few articles focused on the subtypes identification of functional ACAs using imaging modalities. The routine CT images are not only similar in different types of functional ACAs but also do not allow functioning adenomas to be differentiated from non-functioning adenomas, therefore providing merely limited diagnostic value (22).

Obviously, it is of great significance for non-invasive differential diagnosis of ACAs, while discriminating between CPA and APA is still a clinical challenge. In this study, we adopted advanced radiomics to multiphase adrenal CT and constructed machine learning model for classification diagnosis of adrenal adenoma, aiming to investigate whether certain multiparametric CT radiomics can facilitate distinguishing CPA from APA. We also investigated and compared the discrimination performance of different combinations of feature selection and machine learning algorithms in this task.

The differences of functional adrenal adenomas between CPA and APA on conventional CT images were compared. First, there was a certain difference in tumor size between the two groups, and in general, CPA was larger than APA. This may be related to the origination of the tumor tissue. The cells of the zona fasciculata and the zona glomerulosa of the adrenal cortex are responsible for producing cortisol and aldosterone, respectively. Histology shows that the zona fasciculata in the adrenal cortex occupies a larger area than zona glomerulosa; the former is the thickest zonas making up 50% of the cortex, and the latter accounts for around 15% of the thickness of the cortex (23). Next, the mean CT attenuation of CPA on precontrast CT image is higher than that of APA. The adrenal adenomas were composed of different proportions of clear cells and compact cells. APA is mainly composed of a large number of clear cells (lipid-rich) with increased mounts of lipofuscin in the cytoplasm arranged in irregular patches or strips, leading to CT attenuation similar to fat. While CPA mainly presents with granule cell tumors, and the cells are densely arranged in small mesh or fasciculate patterns, with cell cords exhibiting sinus gap shapes and blood sinus, leading to CT attenuation close to soft tissue (24). Lastly, CPA was more likely to develop the ipsilateral or contralateral adrenocortical atrophy than APA. This is associated with atrophy of the non-tumorous cortex due to the negative feedback–suppression effects of the hypothalamic–pituitary axis in CPA. In contrast, the non-tumorous adrenal cortex is not atrophic in glands harboring an APA (24). These findings were basically consistent with previous radiological and pathological reports (7, 24, 25). Although it is still insufficient to distinguish the two tumors on conventional CT image, it may give radiomics the possibility to extract more correlated quantitative features for improving decision support.

CT-based radiomics providing a non-invasive and low-cost analysis technique for tumor property evaluation based on image data has been widely applied (26). The radiomics-based machine learning model can analyze and process CT images in the gray level as well as individual level (27). In the training stage, it is capable of learning from experiential data and hence could discover the general trend of those (priori knowledge). In the test stage, based on the discovered priori knowledge, the model could automate and improve prediction and classification of unknown data effectively, as well as provide the diagnostic information for the individual (28). Until now, the study on the application of radiomics-based computer-aided framework to differential diagnosis between CPA and APA has not been reported. To our knowledge, this is the first study that provides a comprehensive difference quantification of adenomas using radiomics features and gives us new insights for differentiating CPA and APA using machine learning.

In our study, the appropriate feature selection strategies such as ICC analysis, LASSO, and PCA were addressed to enhance the repeatability of radiomics features and improve the classification process by reducing overfitting of models (29). This study evaluated diagnostic capabilities of radiomics features and put much emphasis on the comparison of different machine learning models, because the computational models with high accuracy, reliability, and efficiency of prediction and prognosis are vital factors driving the success of radiomics (18). Radiomics features as imaging biomarkers are emerging and need to be studied and validated prospectively when served in the differential diagnosis of various diseases (30). Our study proposed a radiomics-based machine learning framework to characterize the differences of CT images from the patients with CPA and APA, which could achieve a satisfying clinical outcome. This contributes to simplify the complex diagnostic procedures by voiding the multifarious clinical examinations.

We studied a total of 627 radiomic features extracted from plain scan, arterial phase, and venous phase CT images, including 4 geometric features, 9 first-order statistical features, 40 texture features, and 156 wavelet features in each phase. The 24 radiomic features that differed significantly between the two groups were selected for a radiomics signature. A nomogram that combined radiomic signature with the clinic–radiological features (age, gender, and tumor size, mean CT attenuation, and adrenocortical atrophy) improved the differentiation accuracy in the training and test cohorts. The concept underlying the radiomics process is that both morphological and functional clinical images contain qualitative and quantitative information, which may reflect the underlying tissue-level features, in line with pathological findings (31). Previous studies have reported there were subtle structural and pathological differences between APA and CPA, which had different proportions composed of clear cells (lipid rich), compact cells (lipid poor), cell arrangement, and blood sinus, the same as previously discussed (23, 24). APA cells contained mitochondria with lamellar-type or plate-like cristae, whereas CPA cells contained mitochondria with tubulovesicular cristae (24). Previous studies have indicated that texture analysis and radiomics features were linked with microenvironment heterogeneity within tumors. Quantitative histologic analysis revealed that intratumoral immune cell infiltration was more pronounced in CPAs than in APAs, and the vascular density was also significantly higher in CPAs (32).

The limitation of our work also exists. First, radiomics features are partly associated with VOI segmentations. This study was based on the radiologist-annotated features. Although a high interobserver agreement as well as an excellent feature repeatability has been achieved, it can be subjected to interobserver or intraobserver variability. Automatic or semiautomatic lesion segmentation methods that capture lesions more accurately can be explored in the future. Second, although a prospective study for collecting new cases is still ongoing by our group to increase the sample volume, the low incidence of ACAs determines that a small sample size was used in current research. Third, all the patients were from a single center. Although cross-validation is used for model evaluation, the model may perform differently if multicenter datasets with different parameters are used. Next, a multicenter large-scale data from different institutions should be involved and deep learning could be employed to enhance stability and discrimination performance of model. Future work should extend radiomics to other adrenal tumors such as distinguishing between functional and non-functional adenomas and detecting the nature of adrenal incidentaloma.

## Conclusions

In summary, we have preliminarily investigated the performance of multiparametric CT radiomics-based machine learning model for differentiating CPA from APA. The proposed radiomics analytic framework presents an encouraging result in differential diagnosis between those than conventional imaging techniques. This method may provide a non-invasive and economic approach to facilitate the clinical decision-making in some special conditions such as atypical clinical symptom or hormone secretion and localize responsible lesion in bilateral or multiple tumors.

## Data Availability Statement

All datasets generated for this study are included in the article/Supplementary Material.

## Ethics Statement

The studies involving human participants were reviewed and approved by the institutional review board of the First Affiliated Hospital of Chongqing Medical University. Written informed consent for participation was not required for this study in accordance with the national legislation and the institutional requirements.

## Author Contributions

HY and YZhe: conceptualization and writing—review and editing. YZhe: methodology, software, and validation. LX and YZho: data acquisition. YZhe, HY, and XL: writing—original draft preparation. All authors: contributed to the article and approved the submitted version.

## Conflict of Interest

The authors declare that the research was conducted in the absence of any commercial or financial relationships that could be construed as a potential conflict of interest.

## Abbreviations

ACA: Adrenocortical adenomas
APA: Aldosterone-producing adenoma
CPA: Cortisol-producing adenoma
PA: Primary hyperaldosteronism
ROI: Region of interest
VOI: Volume of interest
ROC: Receiver operating characteristic
AUC: The area under the ROC curve
LR: Logistic regression
SVM: Support vector machine
PCA: Principal component analysis
LASSO: Least absolute shrinkage and selection operator
ICC: Intraclass correlation coefficient.
