# Supplementary material for: A Preliminary Study for Distinguish Hormone-Secreting Functional Adrenocortical Adenoma Subtypes Using Multiparametric CT Radiomics-Based Machine Learning Model and Nomogram
Source: Front Oncol. 2020 Sep 29;10:570502. doi: 10.3389/fonc.2020.570502 (PMC7552922; doi:10.3389/fonc.2020.570502)
Supplement: Supplementary file 1 [file Presentation_1.pdf]

Supplementary Information

The details of radiomics features used in this study.

A total of 627 quantitative radiomics features were extracted from the complete unenhanced, arterial and portal phase CT images, which are divided in four groups as follows: (i) intensity (N=9), (ii) shape (N=4), (iii) texture (N=40) and (iv) wavelet features (N=156).

Intensity features: they describe the distribution of voxel intensities within the MRI image through commonly used and basic metrics. To analyze the spatial distribution of the pixels' hue matrix and extract static features of an images [1].

Table S1 List of intensity features used in this study.

Category (Number of Features)	Feature	Computational formula
Intensity (9)	Variance	$variance = \sigma^2 = \frac{1}{N}\sum_{i=1}^{N}(\mathbf{X}(i) - \bar{X})^2$
	Skewness	$skewness = \frac{\mu_3}{\sigma^3} = \frac{\frac{1}{N}\sum_{i=1}^{N}(\mathbf{X}(i) - \bar{X})^3}{\left(\sqrt{\frac{1}{N}\sum_{i=1}^{N}(\mathbf{X}(i) - \bar{X})^2}\right)^3}$
	Mean	$mean = \bar{X} = \frac{1}{N}\sum_{i=1}^{N}\mathbf{X}(i)$
	Maximum	*The maximum gray level intensity encountered within the ROI*
	Range	$rang = \max(X) - \min(X)$
	Uniformity	$uniformity = \sum_{i=1}^{N_l} p(i)^2$
	Energy	$energy = \sum_{i=1}^{N}\mathbf{X}(i)^2$
	Entropy	$entropy = -\sum_{i=1}^{N_l} p(i)\log_2(p(i) + \epsilon)$
	Kurtosis	$kurtosis = \frac{\mu_4}{\sigma^4} = \frac{\frac{1}{N}\sum_{i=1}^{N}(\mathbf{X}(i) - \bar{X})^4}{\left(\frac{1}{N}\sum_{i=1}^{N}(\mathbf{X}(i) - \bar{X})^2\right)^2}$

Note: X is a set of N voxels representing the voxels included in the ROI. P(i) is the first

order histogram of X with N_l discrete intensity levels, p(i) the normalized first order histogram. ε is an arbitrarily small positive constant.

Shape features: they describe the three-dimensional size and shape of the tumor region [2].

Table S2 List of shape features used in this study.

Category (Number of Features)	Feature	Computational formula
Shape (4)	Asphericity	$asphericity = \left(\frac{1}{36\pi}\frac{A^3}{V^2}\right)^{\frac{1}{3}} - 1$
	Sphericity	$sphericity = \frac{\pi^{\frac{1}{3}}(6V)^{\frac{2}{3}}}{A} = \frac{(36\pi V^2)^{\frac{1}{3}}}{A}$
	Surface to volume ratio	The ratio of surface to volume
	Elongation	$Elongation = \frac{\lambda_{\text{longest}}}{\lambda_{\text{intermediate}}}$

Note: V is the volume of the VOI and A is the surface area of the VOI

Textural features: they reflect the homogeneity phenomenon of images and the arrangement of properties that change slowly or periodically on the body surface. The textural features mainly included gray-level co-occurrence (GLCM), gray-level run-length (GLRLM), gray-level size-zone (GLSZM), gray-level distance-zone (GLDZM) and neighborhood gray tone difference (NGTDM). GLCM is the matrix function that describes the distance and angle of each pixel, which can reflect integrated information regarding the direction, interval, amplitude, and frequency of images. GLRLM quantify gray level runs in an image. GLSZM quantifies gray level zones in an image. NGTDM represents the summation of the gray-level differences between all voxels with gray-level i and the average gray-level of their 26-connected neighbors in 3D space [3].

Table S3 List of texture features used in this study.

Category (Number of Features)	Feature	Computational formula
Texture	Energy	$energy = \sum_{i=1}^{N_g}\sum_{j=1}^{N_g}(p(i,j))^2$
	Contrast	$contrast = \sum_{i=1}^{N_g}\sum_{j=1}^{N_g}(i-j)^2 p(i,j)$
	Correlation	$correlation = \frac{\sum_{i=1}^{N_g}\sum_{j=1}^{N_g} p(i,j)ij - \mu_x(i)\mu_y(j)}{\sigma_x(i)\sigma_y(j)}$
	Homogeneity	$homogeneity\ 1 = \sum_{i=1}^{N_g}\sum_{j=1}^{N_g}\frac{p(i,j)}{1+\lvert i-j\rvert}$
	Sum Variance	$sum\ variance = \sum_{k=2}^{2N_g}(k-SE)^2 p_{x+y}(k)$
	Sum Average	$sum\ average = \sum_{k=2}^{2N_g} p_{x+y}(k)k$
	Sum Entropy	$sum\ entropy = \sum_{k=2}^{2N_g} p_{x+y}(k)\log_2(p_{x+y}(k)+\epsilon)$
	Dissimilarity	$dissimilarity = \sum_{i=1}^{N_g}\sum_{j=1}^{N_g}\lvert i-j\rvert p(i,j)$
	Autocorrelation	$autocorrelation = \sum_{i=1}^{N_g}\sum_{j=1}^{N_g} p(i,j)ij$
	Short Run Emphasis (SRE)	$SRE = \frac{\sum_{i=1}^{N_g}\sum_{j=1}^{N_r}\frac{\mathbf{P}(i,j\|\theta)}{i^2}}{\sum_{i=1}^{N_g}\sum_{j=1}^{N_r}\mathbf{P}(i,j\|\theta)}$
	Long Run Emphasis (LRE)	$LRE = \frac{\sum_{i=1}^{N_g}\sum_{j=1}^{N_r}\mathbf{P}(i,j\|\theta)j^2}{\sum_{i=1}^{\square_g}\sum_{j=1}^{N_r}\mathbf{P}(i,j\|\theta)}$
	Gray-level Non-uniformity (GLN)	$GLN = \frac{\sum_{i=1}^{N_g}\left(\sum_{j=1}^{N_r}\mathbf{P}(i,j\|\theta)\right)^2}{\sum_{i=1}^{N_g}\sum_{j=1}^{N_\square}\mathbf{P}(i,j\|\theta)}$
	Run-Length Non-uniformity (RLN)	$RLN = \frac{\sum_{j=1}^{N_r}\left(\sum_{i=1}^{N_g}\mathbf{P}(i,j\|\theta)\right)^2}{\sum_{i=1}^{N_g}\sum_{j=1}^{N_r}\mathbf{P}(i,j\|\theta)}$
	Run Percentage (RP)	$RP = \sum_{i=1}^{N_g}\sum_{j=1}^{N_r}\frac{\mathbf{P}(i,j\|\theta)}{N_p}$
	Low Gray-level Run Emphasis (LGRE)	$LGLRE = \frac{\sum_{i=1}^{N_g}\sum_{j=1}^{N_r}\frac{\mathbf{P}(i,j\|\theta)}{i^2}}{\sum_{i=1}^{N_g}\sum_{j=1}^{N_r}\mathbf{P}(i,j\|\theta)}$
	High Gray-level Run Emphasis (HGRE)	$HGLRE = \frac{\sum_{i=1}^{N_g}\sum_{j=1}^{N_r}\mathbf{P}(i,j\|\theta)i^2}{\sum_{i=1}^{N_g}\sum_{j=1}^{N_r}\mathbf{P}(i,j\|\theta)}$

	Short Run Low Gray-level Emphasis (SRLGE)	$SRLGLE = \frac{\sum_{i=1}^{N_g}\sum_{j=1}^{N_r}\frac{\mathbf{P}(i,j\|\theta)}{i^2j^2}}{\sum_{i=1}^{N_g}\sum_{j=1}^{N_r}\mathbf{P}(i,j\|\theta)}$
	Short Run High Gray-level Emphasis (SRHGE)	$SRHGLE = \frac{\sum_{i=1}^{N_g}\sum_{j=1}^{N_r}\frac{\mathbf{P}(i,j\|\theta)i^2}{j^2}}{\sum_{i=1}^{N_g}\sum_{j=1}^{N_r}\mathbf{P}(i,j\|\theta)}$
	Long Run Low Gray-level Emphasis (LRLGE)	$LRLGLRE = \frac{\sum_{i=1}^{N_g}\sum_{j=1}^{N_r}\frac{\mathbf{P}(i,j\|\theta)j^2}{i^2}}{\sum_{i=1}^{N_g}\sum_{j=1}^{N_r}\mathbf{P}(i,j\|\theta)}$
	Long Run High Gray-level Emphasis (LRHGE)	$LRHGLRE = \frac{\sum_{i=1}^{N_g}\sum_{j=1}^{N_r}\mathbf{P}(i,j\|\theta)i^2j^2}{\sum_{i=1}^{N_g}\sum_{j=1}^{N_r}\mathbf{P}(i,j\|\theta)}$
	Gray-level Variance (GLV)	$GLV = \sum_{i=1}^{N_g}\sum_{j=1}^{N_r}(i-\mu_g)^2 p_n(i,j)$
	Run-Length Variance (RLV)	$RLV = \sum_{i=1}^{N_g}\sum_{j=1}^{N_r}(j-\mu_r)^2 p_n(i,j)$
	Small Zone Emphasis (SZE)	$SAE = \frac{\sum_{i=1}^{N_g}\sum_{j=1}^{N_s}\frac{\mathbf{P}(i,j)}{j^2}}{\sum_{i=1}^{N_g}\sum_{j=1}^{N_s}\mathbf{P}(i,j)}$
	Large Zone Emphasis (LZE)	$LAE = \frac{\sum_{i=1}^{N_g}\sum_{j=1}^{N_s}\mathbf{P}(i,j)j^2}{\sum_{i=1}^{N_g}\sum_{j=1}^{N_s}\mathbf{P}(i,j)}$
	Gray-level Non-uniformity (GLN)	$GLN = \frac{\sum_{i=1}^{N_g}\left(\sum_{j=1}^{N_s}\mathbf{P}(i,j)\right)^2}{\sum_{i=1}^{N_g}\sum_{j=1}^{N_s}\mathbf{P}(i,j)}$
	Zone-Size Non-uniformity (ZSN)	$SZN = \frac{\sum_{j=1}^{N_s}\left(\sum_{i=1}^{N_g}\mathbf{P}(i,j)\right)^2}{\sum_{i=1}^{N_g}\sum_{j=1}^{N_s}\mathbf{P}(i,j)}$
	Zone Percentage (ZP)	$ZP = \sum_{i=1}^{N_g}\sum_{j=1}^{N_s}\frac{\mathbf{P}(i,j)}{N_p}$
	Low Gray-level Zone Emphasis (LGZE)	$LGZE = \sum_{i=1}^{N_g}\sum_{j=1}^{L_z}\frac{p(i,j)}{i^2}$
	High Gray-level Zone Emphasis (HGZE)	$HGLZE = \frac{\sum_{i=1}^{N_g}\sum_{j=1}^{N_s}\mathbf{P}(i,j)i^2}{\sum_{i=1}^{N_g}\sum_{j=1}^{N_s}\mathbf{P}(i,j)}$
	Small Zone Low Gray-level Emphasis (SZLGE)	$SALGLE = \frac{\sum_{i=1}^{N_g}\sum_{j=1}^{N_s}\frac{\mathbf{P}(i,j)}{i^2j^2}}{\sum_{i=1}^{N_g}\sum_{j=1}^{N_s}\mathbf{P}(i,j)}$
	Small Zone High Gray-level Emphasis (SZHGE)	$SAHGLE = \frac{\sum_{i=1}^{N_g}\sum_{j=1}^{N_s}\frac{\mathbf{P}(i,j)i^2}{j^2}}{\sum_{i=1}^{N_g}\sum_{j=1}^{N_s}\mathbf{P}(i,j)}$
	Large Zone Low Gray-level Emphasis (LZLGE)	$LALGLE = \frac{\sum_{i=1}^{N_g}\sum_{j=1}^{N_s}\frac{\mathbf{P}(i,j)j^2}{i^2}}{\sum_{i=1}^{N_g}\sum_{j=1}^{N_s}\mathbf{P}(i,j)}$

	Large Zone High Gray-level Emphasis (LZHGE)	$LAHGLE = \frac{\sum_{i=1}^{N_g}\sum_{j=1}^{N_s}\mathbf{P}(i,j)i^2j^2}{\sum_{i=1}^{N_g}\sum_{j=1}^{N_s}\mathbf{P}(i,j)}$
	Gray-level Variance (GLV)	$GLV = \frac{1}{N_g \times L_z}\sum_{i=1}^{N_g}\sum_{j=1}^{L_z}(i\,p(i,j) - \mu_i)^2$
	Zone-Size Variance (ZSV)	$ZSV = \frac{1}{N_g \times L_z}\sum_{i=1}^{N_g}\sum_{j=1}^{L_z}(j\,p(i,j) - \mu_j)^2$
	Coarseness	$coarseness = \frac{1}{\varepsilon + \sum_{i=i}^{N_g} p(i)s(i)}$
	Contrast	$contrast = \left(\frac{1}{N_p(1-N_p)}\sum_{i=1}^{N_g}\sum_{j=1}^{N_g} p(i)p(j)(i-j)^2\right)\left(\frac{1}{N_v}\sum_{i=i}^{N_g} s(i)\right)$
	Busyness	$busyness = \frac{\sum_{i=i}^{N_g} p(i)s(i)}{\sum_{i=i}^{N_g}\sum_{j=i}^{N_g}\lvert ip(i) - jp(j)\rvert}$
	Complexity	$complexity = \frac{1}{N_v}\sum_{i=i}^{N_g}\sum_{j=i}^{N_g}\lvert i-j\rvert\frac{p(i)s(i)+p(j)s(j)}{p(i)+p(j)}$
	Strength	$strength = \frac{\sum_{i=i}^{N_g}\sum_{j=i}^{N_g}[p(i)+p(j)](i-j)^2}{\varepsilon + \sum_{i=i}^{N_g} s(i)}$

Wavelet features [4]: The wavelet transform was used to decompose the original images, which can be regarded as a preprocessing prior to feature extraction. By changing the ratio of high-frequency to low-frequency signal in images, wavelet transform increases the information of low-frequency signal. Consider L and H to be a low-pass and high-pass functions respectively, X to be the decomposing image.

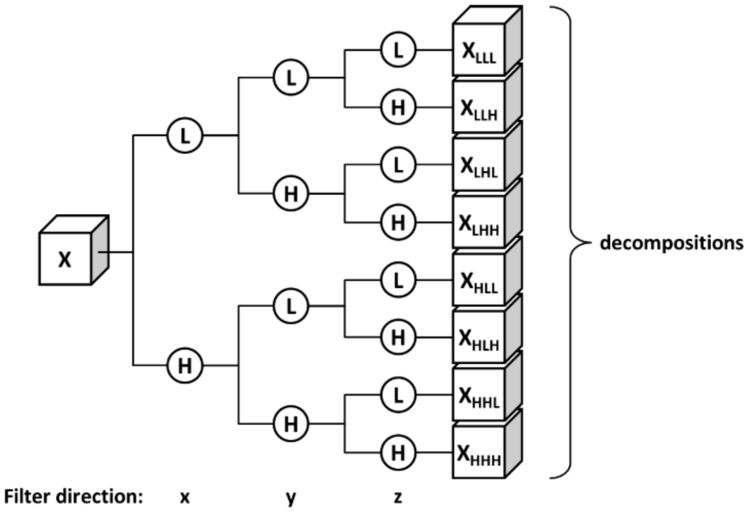

Fig. S1 Schematic of the three-dimensional wavelet transform applied to each CT image [4].

The detailed description of the LASSO method

LASSO is a powerful method for regression with high dimensional predictors. In our study, the LASSO method was combined with Cox proportional hazard regression model for survival analysis. We used the LASSO Cox regression model to select the most important prognostic features from the training dataset. This method minimizes a log partial likelihood subject to the sum of the absolute values of the parameters being bounded by a constant:

$$\hat{\beta} = \text{argmin}\ \ell(\beta), \text{ subject to } \sum|\beta_j| \le s$$

where, $\hat{\beta}$ is the obtained parameters, $\ell(\beta)$ is the log partial likelihood of the Cox proportional hazard regression model, $s > 0$ is a constant [5].

A

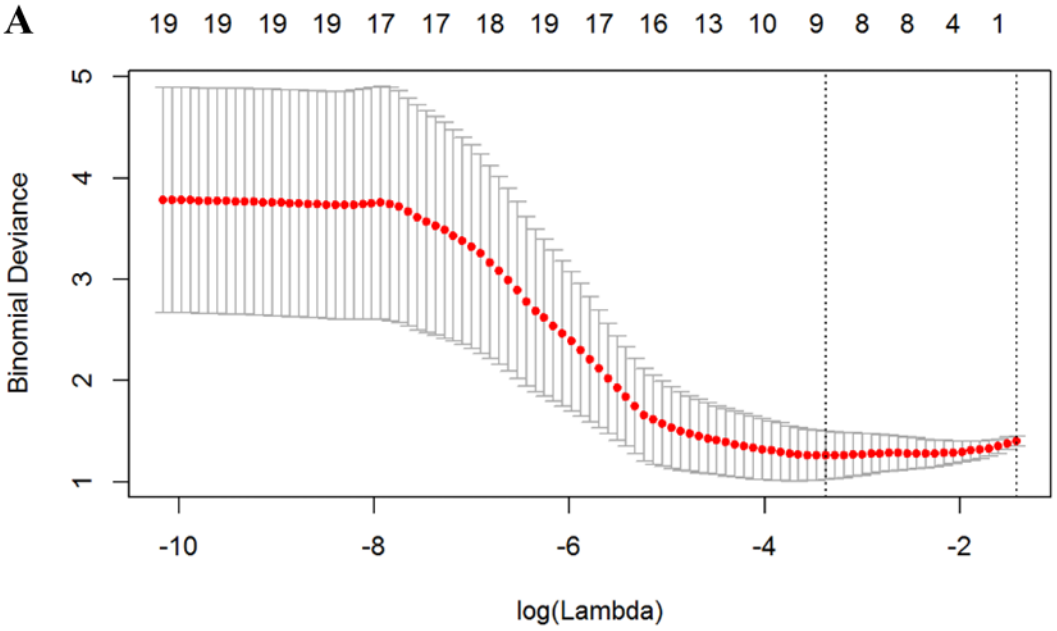

B

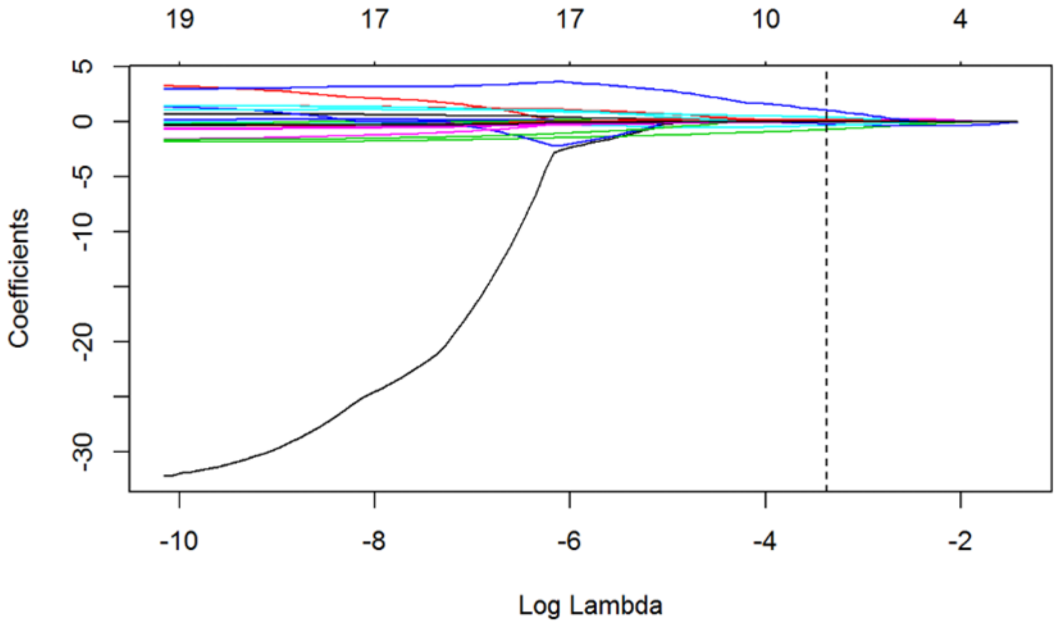

Fig. S2 Radiomics feature selection using the least absolute shrinkage and selection operator (LASSO) logistic regression model. (A) Identification of the optimal penalization coefficient lambda (λ) in the LASSO model used 5-fold cross-validation and the minimum criterion. (B) LASSO coefficient profiles of the all radiomics features.

Table S4 The selective radiomics features with high ICCs

Image phase	Feature	ICC	Feature	ICC
Plain scan	Skewness*	0.815	GLV*	0.861
	SZHGE*	0.844	ZSV	0.903
	SRE*	0.876	Coarseness*	0.812
	LGRE*	0.927		
Arterial	Skewness	0.816	LZHGE*	0.877
	Skewness*	0.901	ZSV	0.859
	RP	0.933	Contrast*	0.815
	HGRE*	0.857	Complexity*	0.808
	LRLGE	0.829	Busyness*	0.803
	LRHGE*	0.818	Wavelet_HL_RLN	0.800

	LZE*	0.925	Wavelet_HL_HGZE*	0.810
	ZP*	0.884	Wavelet_HL_GLV	0.823
	SZLGE	0.863	Wavelet_LL_GLV	0.807
	LZLGE*	0.871	Wavelet_LL_LZE	0.811
	Variance*	0.935	ZSN	0.914
	Uniformity*	0.875	LZLGE*	0.847
Venous	Sphericity	0.865	Complexity*	0.889
	Entropy*	0.874	Wavelet_HL_ Homogeneity*	0.814
	HGRE*	0.825	Wavelet_LL_ Autocorrelation	0.876
	SZE	0.838	Wavelet_LL_ SZHGE	0.880

*: significant difference between APA and CPA

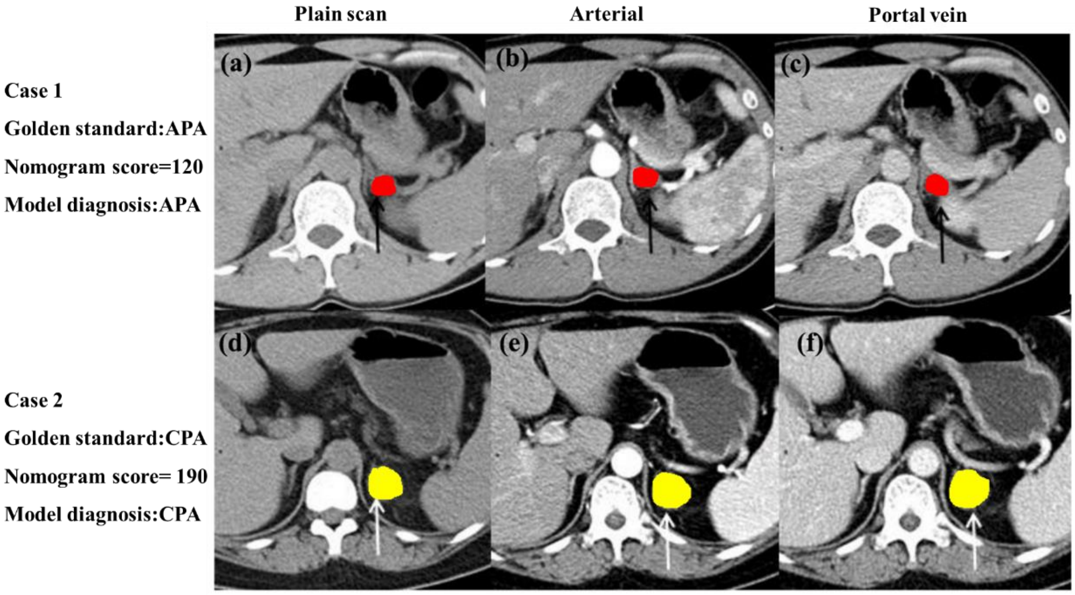

Fig. S3 The illustration of two cases pathologically diagnosed as CPA and APA respectively and the probability values predicted by the nomogram.

Reference:

[1] Thibault G, Angulo J, Meyer F. Advanced statistical matrices for texture characterization: application to cell classification[J]. IEEE Transactions on Biomedical Engineering, 2013, 61(3): 630-637.

[2] da Silva Sousa J R F, Silva A C, de Paiva A C, et al. Methodology for automatic detection of lung nodules in computerized tomography images[J]. Computer methods and programs in biomedicine, 2010, 98(1): 1-14.

[3] Tixier F, Hatt M, Le Rest C C, et al. Reproducibility of tumor uptake heterogeneity characterization through textural feature analysis in 18F-FDG PET[J]. Journal of Nuclear Medicine, 2012, 53(5): 693-700.

[4] Aerts H J W L, Velazquez E R, Leijenaar R T H, et al. Decoding tumour phenotype by noninvasive imaging using a quantitative radiomics approach[J]. Nature communications, 2014, 5(1): 1-9.

[5] Tibshirani R. The lasso method for variable selection in the Cox model[J]. Statistics in medicine, 1997, 16(4): 385-395.
